# Supplementary material for: Ultrahigh-responsivity waveguide-coupled optical power monitor for Si photonic circuits operating at near-infrared wavelengths
Source: Nat Commun. 2022 Dec 9;13:7443. doi: 10.1038/s41467-022-35206-4 (PMC9734153; doi:10.1038/s41467-022-35206-4)
Supplement: Supplementary file 1 — Supplementary Information [file 41467_2022_35206_MOESM1_ESM.pdf]

Supplementary Information

Ultrahigh-responsivity waveguide-coupled optical power monitor for Si photonic circuits operating at near-infrared wavelengths

Takaya Ochiai[1], Tomohiro Akazawa[1], Yuto Miyatake[1], Kei Sumita[1], Shuhei Ohno[1], Stéphane Monfray[2], Frederic Boeuf[2], Kasidit Toprasertpong[1], Shinichi Takagi[1], Mitsuru Takenaka[1*]

[1] Department of Electrical Engineering and Information Systems, The University of Tokyo, 7-3-1 Hongo, Bunkyo-ku, Tokyo 113-8656, Japan,

Phone: +81-3-5841-6733, Fax: +81-3-5841-8564,

[2] STMicroelectronics, 850 Rue Jean Monnet 38920 Crolles, France

*E-mail: takenaka@mosfet.t.u-tokyo.ac.jp

Contents

I. Fabrication procedure for waveguide-coupled InGaAs phototransistor

II. Band structure and electric field distribution

III. Measurement of electron mobility of InGaAs transistor

IV. Propagation loss of the Si waveguide

V. Waveform of modulated light

VI. Measurement of noise equivalent power and specific detectivity

I. Fabrication procedure for waveguide-coupled InGaAs phototransistor

Figure S1 shows the process flow of the waveguide-coupled InGaAs phototransistor. Si rib waveguides were fabricated on a Si-on-insulator (SOI) wafer with a 300-nm-thick Si

layer. The width and the rib height were 400 nm and 150 nm for a single-mode operation at a 1.3 μm wavelength. Grating couplers for the transverse electric (TE) mode were integrated with the Si waveguide for fiber coupling. After forming SiO_2 cladding by chemical vapor deposition (CVD) on the Si waveguide, chemical mechanical polishing (CMP) was carried out for surface planarization. To form the p-Si (acceptor concentration $N_A = 5 \times 10^{17}$ cm^{-3}) and p$^+$-Si ($N_A = 1 \times 10^{20}$ cm^{-3}) regions, boron ions were implanted. Then, an InP epitaxial wafer consisting of a p-type 30-nm-thick $In_{0.53}Ga_{0.47}As$ layer ($N_A = 5 \times 10^{16}$ cm^{-3}) and etch-stop layers was bonded onto the Si waveguide with an Al_2O_3 bonding layer formed by atomic layer deposition (ALD)[1]. After bonding, the InP wafer and the etch-stop layers were selectively removed by wet etching. The 40-nm-thick SiO_2 hard mask formed by CVD was patterned by electron-beam (EB) lithography and inductively coupled plasma (ICP) etching. Then, the InGaAs layer was patterned by wet etching. After removing the SiO_2 hard mask, a 10-nm-thick Al_2O_3 passivation layer was formed by ALD. Finally, the contact windows for source and drain (S/D) were opened, and Ni/Au contact pads as metal S/D[2,3] were formed by EB evaporation and lift-off. The Ni/Au contact pad for the gate was also formed simultaneously. Figure S2 shows a plan-view microscopy image of the fabricated device. The contact pad for the Si waveguide gate was formed away from the S/D region owing to the limitation of the original Si photonic circuit design.

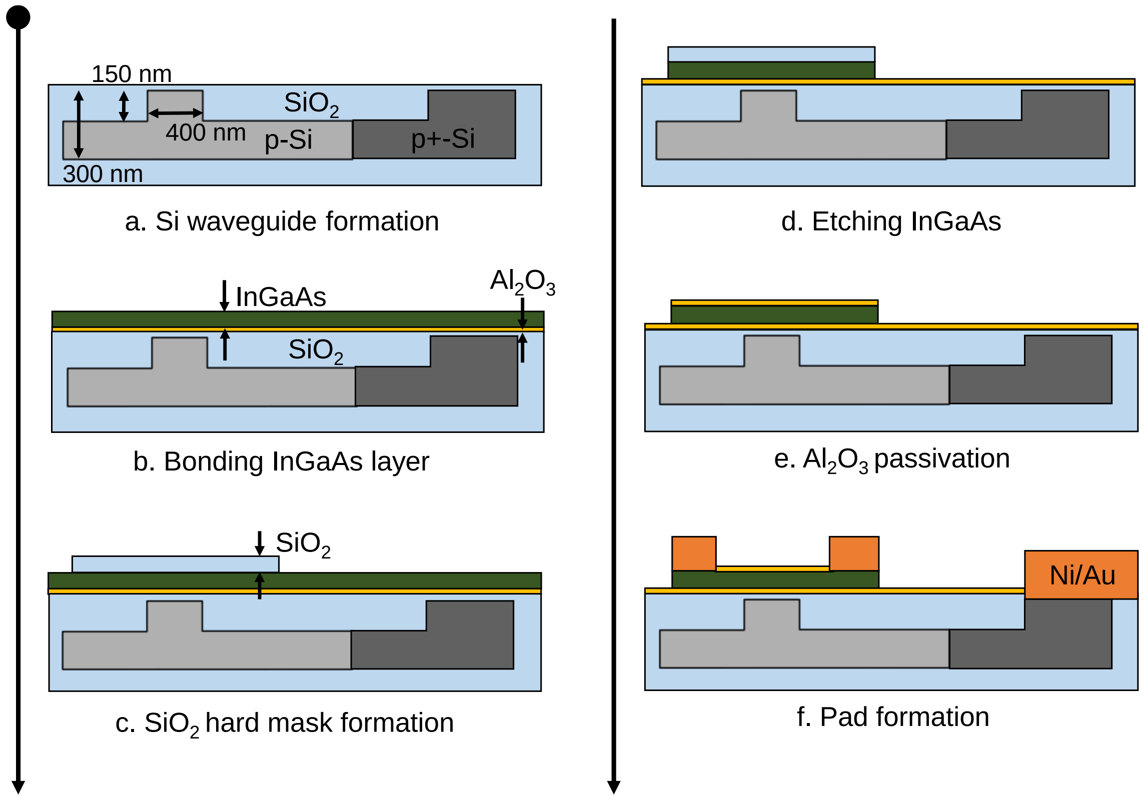

Fig. S1. **Process flow of waveguide-coupled InGaAs phototransistor.** An InGaAs ultrathin membrane is bonded onto a p-type Si waveguide with Al_2O_3 gate dielectric. The Si waveguide acts as a gate electrode, and the Ni/Au metal source and drain are deposited on the InGaAs channel.

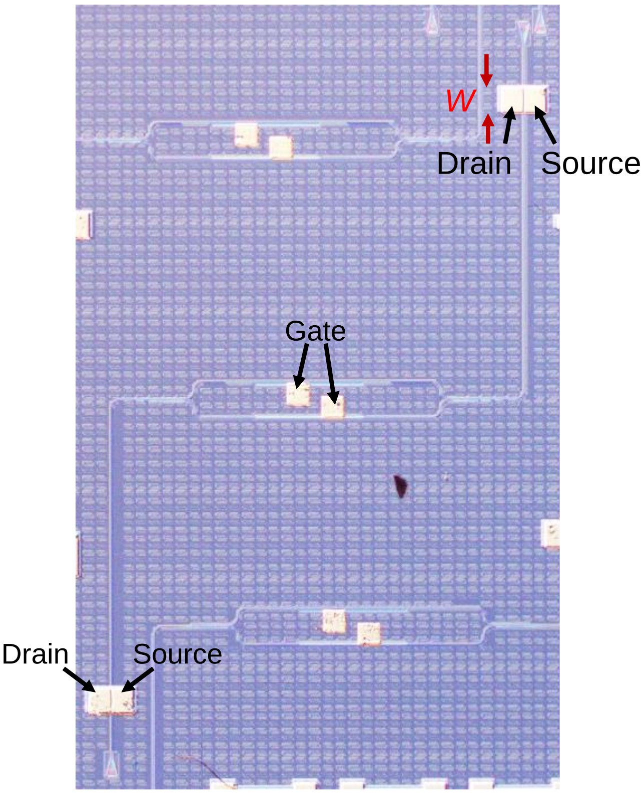

Fig. S2. **Plan-view microscopy image of waveguide-coupled InGaAs phototransistors.**

II. Band structure and electric field distribution

The operation principle of a phototransistor with the metal source and drain is depicted in Fig. S3. Since n-type transistor is considered here, the Schottky contact with large barrier height for holes are assumed. When no light is injected, the transistor is off, resulting in a low drain current. When the transistor channel is irradiated by light, photo-generated holes accumulate in the channel, pushing the conduction band and valence band down. As a result, the transistor turns on, and more drain current flows through the channel. In this way, the photocurrent is amplified through the change in the transistor conduction.

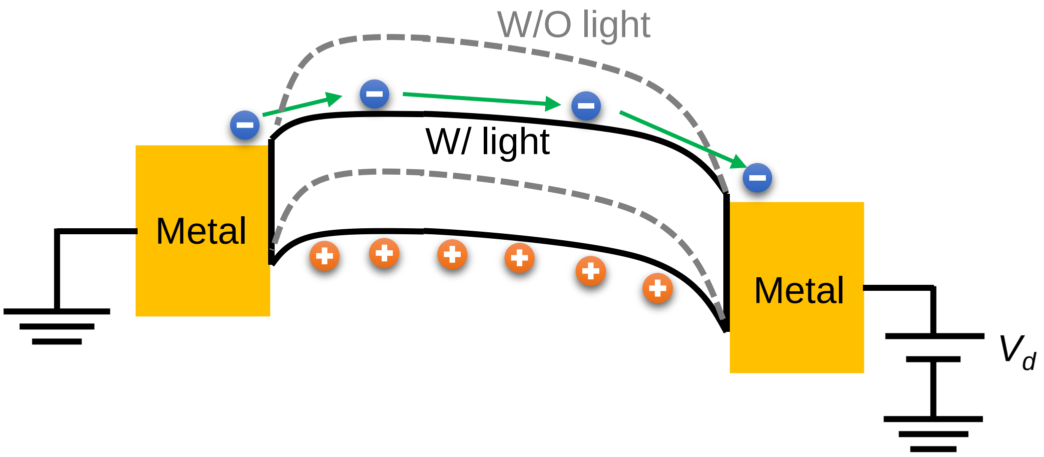

Fig. S3. **Operation principle of a phototransistor with metal source and drain.**

The band diagram of the proposed waveguide-coupled InGaAs phototransistor was simulated using Ansys Lumerical DEVICE when V_d and V_g are 0.5 V and 0 V, respectively, as shown in Fig. S4. Here, the Schottky contact with a barrier height of 0.1 eV for electrons was assumed as the metal source and drain. Here, an n-type InGaAs layer with a doping density of 5×10^{16} cm^{-3} was assumed to represent the negative threshold voltage observed in the experiments. As shown in Fig. S4, the channel under the gate has a potential barrier for electrons that is modulated by light injection. The distributions of the electro-static potential and electric filed are also shown in Fig. S5. Note that the electrical contact to the Si waveguide was set at the bottom of the Si layer due to the limitation of the simulation.

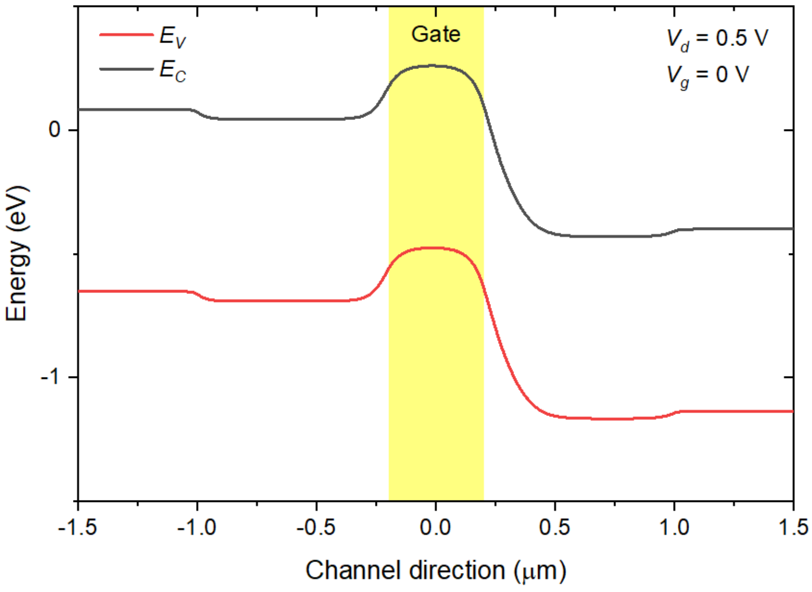

Fig. S4. **Band diagram of waveguide-coupled InGaAs phototransistor across the channel direction.**

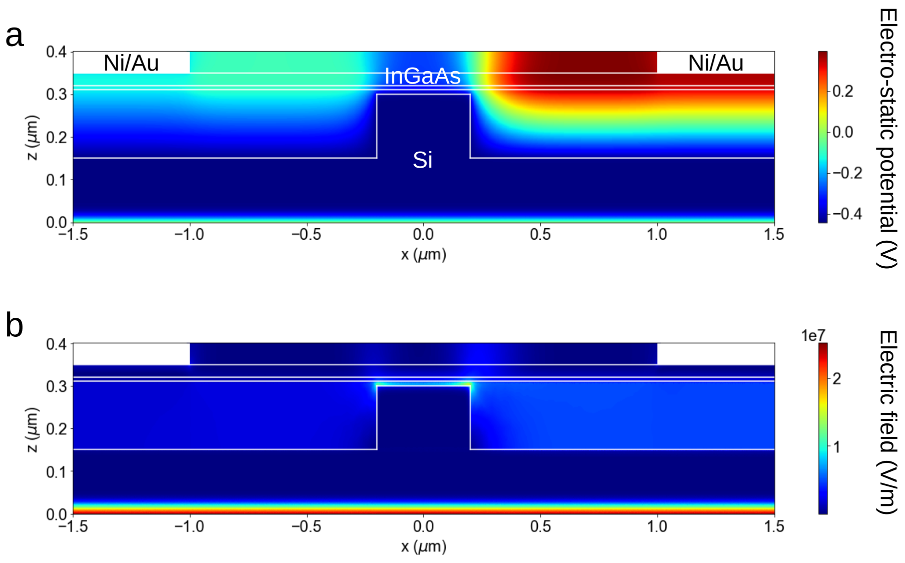

Fig. S5. **Distributions of the electric-static potential and electric field in waveguide-coupled InGaAs phototransistor. a,** Electro-static potential when V_d and V_g are 0.5 V and 0 V , respectively. **b,** Electric field when V_d and V_g are 0.5 V and 0 V , respectively.

III. Measurement of electron mobility of InGaAs transistor

We prepared the InGaAs transistor shown in Fig. S6 for mobility measurement. From the drain current (I_d) – gate voltage (V_g) characteristics in Fig. S7a, we extracted the field-effect electron mobility μ_{FE} as

$$\mu_{FE} = \frac{dI_d}{dV_g}\frac{L}{W}\frac{1}{C_{ox}V_d}, \quad (1)$$

where L is the channel length, W is the channel width, and C_{ox} is the gate capacitance. Here, L was 20 μm, and W was 30 μm. C_{ox} was calculated to be 0.298 μF/cm^2 by taking into account the 6-nm-thick SiO_2 and 10-nm-thick Al_2O_3 layers. Figure S7b shows the extracted μ_{FE} as a function of V_g. The peak value of the electron mobility was approximately 608 cm^2/Vs. Note that there is room for improvement in electron mobility through the process optimization due to the high electron mobility of InGaAs[3].

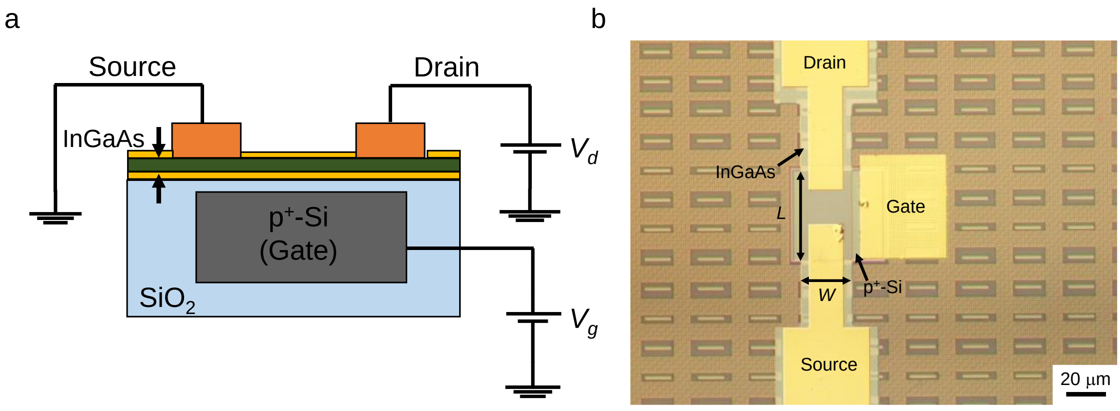

Fig. S6. **InGaAs transistor for mobility measurement. a,** Cross-sectional schematic of the InGaAs transistor. **b,** Plan-view microscopy image of the InGaAs transistor.

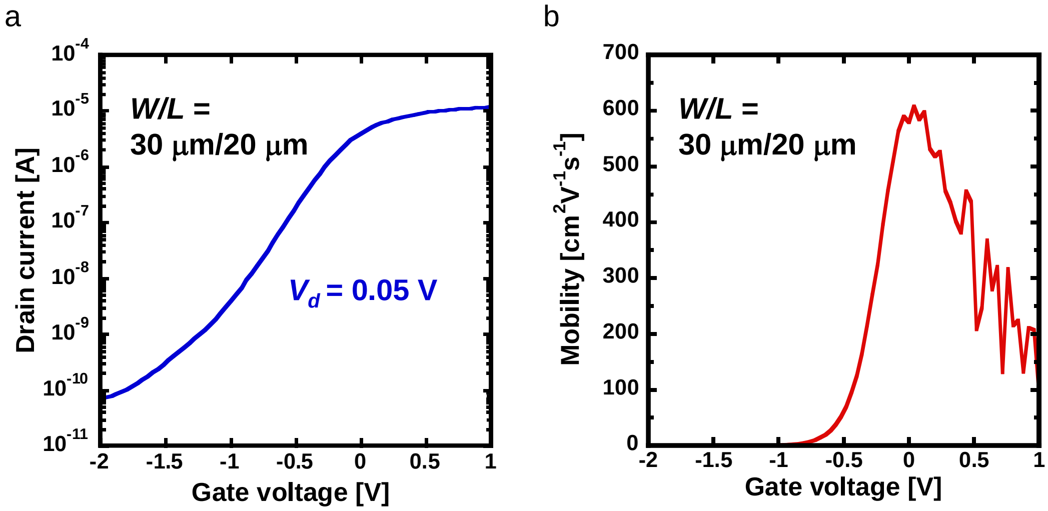

Fig. S7. **Electrical characteristics of the InGaAs transistor. a,** Drain current (I_d) – gate voltage (V_g) characteristics. **b,** Field-effect electron mobility as a function of gate voltage.

IV. Propagation loss of the Si waveguide

We evaluated the propagation loss of the Si waveguide and the coupling loss of the grating coupler at a wavelength of 1305 nm to obtain the optical power injected into the phototransistor. We prepared the Si waveguide of various waveguide lengths from 1296 μm to 12251 μm, as shown in Fig. S8a. A continuous wave (CW) light from a tunable laser was coupled into the Si waveguide through the grating coupler, and the output power from the Si waveguide was coupled again to a single-mode fiber through another grating coupler. The output power was measured using an InGaAs optical power monitor. The polarization of the input light was tuned to the transverse-electric mode using a polarizer. A variable attenuator was also inserted between the tunable laser and the polarizer to adjust the input power. The insertion loss from the tunable laser to the power meter without the device under test was measured to be 5.2 dB. Figure S8b shows the

transmission spectra of the Si waveguide of various waveguide lengths. From the results in Fig. S8b, the propagation loss of the Si waveguide and the coupling loss of the grating coupler were extracted to be 2.14 dB/cm and 7.5 dB, respectively, as shown in Fig. S8c. Since the length of the Si waveguide from the grating coupler to the PD is 95 μm, the total insertion loss, which is dominated by the coupling loss of the grating coupler, is 7.52 dB.

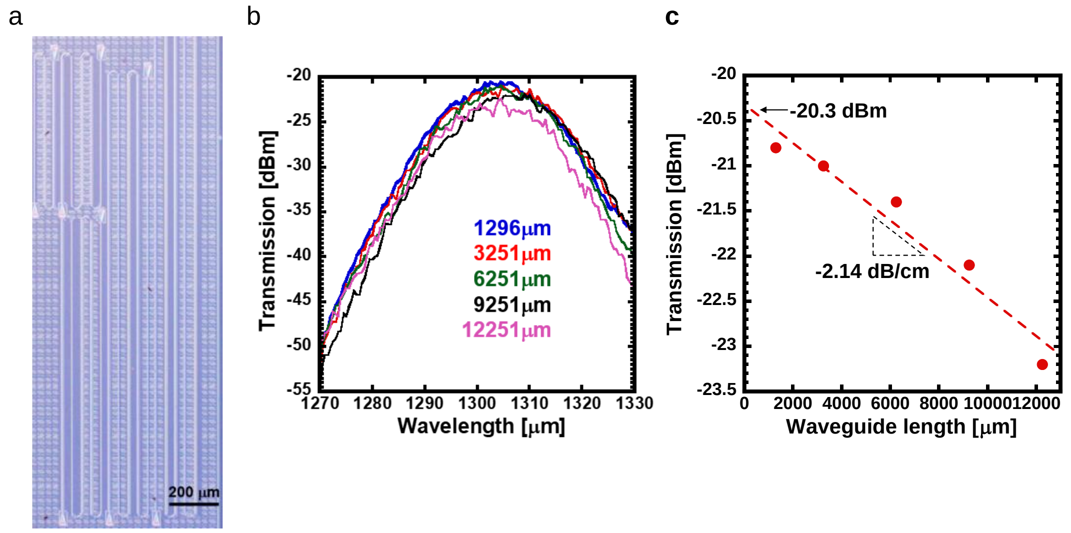

Fig. S8. **Measurement of the propagation loss and coupling loss. a,** Plan-view microscopy image of the Si waveguides of various waveguide lengths for loss measurement. **b,** Transmission spectra of the Si waveguide of various waveguide lengths. **c,** Transmission as a function of waveguide length.

V. Waveform of the modulated light

The tunable laser was modulated with a 100-Hz electrical square signal from a function generator to generate modulated light for time response measurement. To confirm the rise time and fall time of light modulation, the waveform of the modulated light was measured

using a photodetector with the response time of approximately 20 ns, as shown in Fig. S9. The rise time and fall time were 400 ns and 100 ns, respectively.

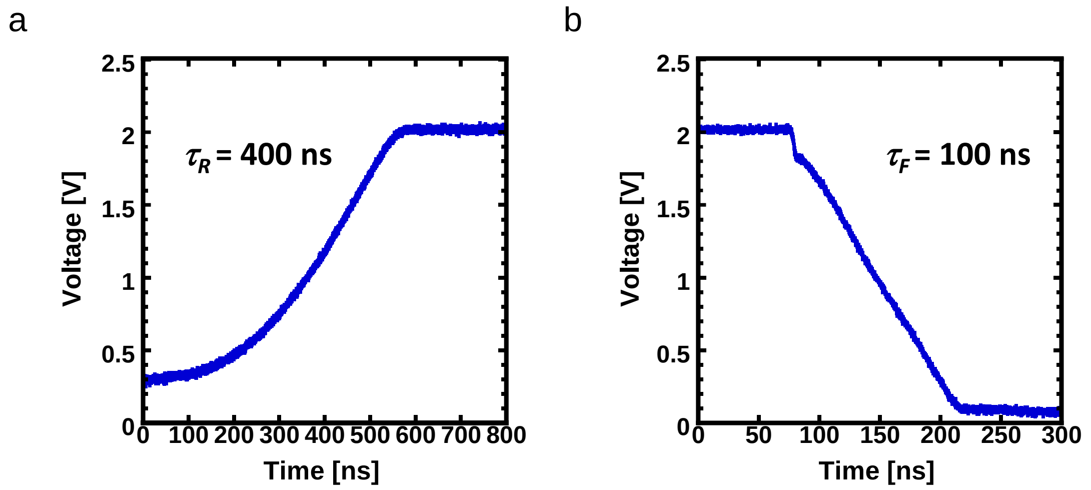

Fig. S9. **Waveform of the modulated light. a,** Rising edge. **b,** Falling edge.

VI. Measurement of noise equivalent power and specific detectivity

The noise power density spectrum of an InGaAs phototransistor was evaluated by the Fourier transform of the dark current waveform measured using a waveform generator/fast measurement unit (Agilent, B1530A) of a semiconductor device analyzer (Agilent Technologies, B1500A). Figure S10 shows the measured noise power density spectrum when V_d and V_g were 0.5 V and 1.0 V, respectively. As shown in Fig. S10, the noise power density was proportional to $1/f^2$, where f is a frequency. According to the method described in Ref. 4, the noise equivalent power (NEP) was extracted by integrating the noise power density from 0.1 Hz to 10 kHz. The specific detectivity was then obtained from the NEP, where the area of the phototransistor was the product of the waveguide width (0.4 μm) and InGaAs length (30 μm).

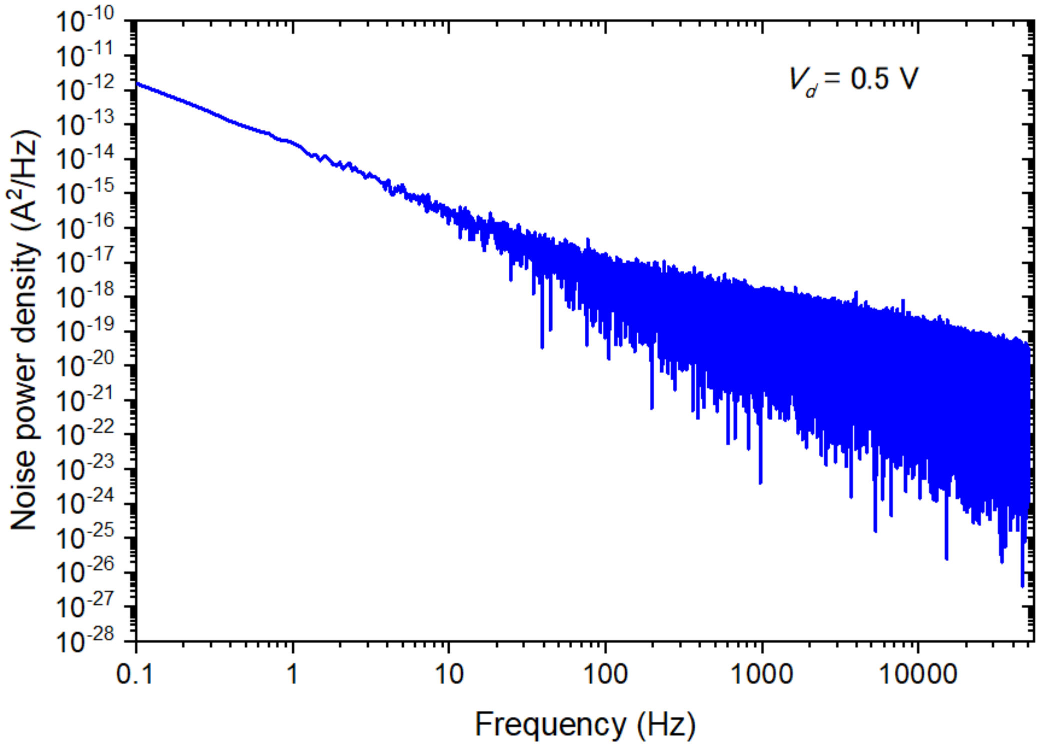

Fig. S10. **Measured noise power density of waveguide-coupled InGaAs phototransistor.** V_d and V_g was 0.5 V and 1.0 V, respectively.

References

1. Han, J. H., Boeuf, F., Fujikata, J., Takahashi, S., Takagi, S. & Takenaka, M. Efficient low-loss InGaAsP/Si hybrid MOS optical modulator. *Nat. Photonics* **11**, 486–490 (2017).

2. Zhang, X. *et al.* $In_{0.7}Ga_{0.3}As$ channel n-MOSFET with self-aligned Ni–InGaAs source and drain. *Electrochemical and Solid-State Letters* **14**, H60 (2010).

3. Kim, S. H. *et al.* High Performance extremely thin body InGaAs-on-insulator metal–oxide–semiconductor field-effect transistors on Si substrates with Ni–InGaAs metal

source/drain. *Appl. Phys. Express* **4**,114201 (2011).

4. Weng, W. Y., Chang, S. J., Hsu, C. L. & Hsueh, T. J. A ZnO-nanowire phototransistor prepared on glass substrates. *ACS Appl. Mater. Interfaces* **3**, 162–166 (2011).
